# Down Syndrome and Autoimmune Disease

**DOI:** 10.1007/s12016-024-08996-2

**Published:** 2024-06-24

**Authors:** Brian Hom, Natalie K. Boyd, Benjamin N. Vogel, Nicole Nishimori, Mellad M. Khoshnood, Saba Jafarpour, Deepti Nagesh, Jonathan D. Santoro

**Affiliations:** 1 Keck School of Medicine at the University of Southern California, Los Angeles, CA USA; 2 Division of Neurology, Department of Pediatrics, Children’s Hospital Los Angeles, 4650 Sunset Blvd, MS82, Los Angeles, CA90027 USA; 3 Department of Neurology, Keck School of Medicineat the, University of Southern California , Los Angeles, CA USA

**Keywords:** Down syndrome, Autoimmunity, Chromosome 21, Immune dysregulation

## Abstract

Down syndrome is the most common genetic cause of intellectual disability and has previously been associated with a variety of autoimmune disorders affecting multiple organ systems. The high prevalence of autoimmune disease, in conjunction with other inflammatory and infectious diseases, in this population suggests an intrinsic immune dysregulation associated with triplication of chromosome 21. Emerging data on the role of chromosome 21 in interferon activation, cytokine production, and activation of B-cell mediated autoimmunity are emerging hypotheses that may explain the elevated prevalence of autoimmune thyroid disease, celiac disease, type I diabetes, autoimmune skin disease, and a variety of autoimmune neurologic conditions. As the life expectancy for individuals with Down syndrome increases, knowledge of the epidemiology, clinical features, management and underlying causes of these conditions will become increasingly important. Disorders such as Hashimoto’s thyroiditis are prevalent in between 13 and 34% of individuals with Down syndrome but only 3% of the neurotypical population, a pattern similarly recognized in individuals with Celiac Disease (5.8% v 0.5–2%), alopecia areata (27.7% v. 2%), and vitiligo (4.4% v. 0.05–1.55%), respectively. Given the chronicity of autoimmune conditions, early identification and management can significantly impact the quality of life of individuals with Down syndrome. This comprehensive review will highlight common clinical autoimmune conditions observed in individuals with Down syndrome and explore our current understanding of the mechanisms of disease in this population.

## Introduction

Down syndrome (DS) is one of the most common genetic disorders and causes of intellectual disability, with an estimated prevalence of 1 in 700 live births [1]. DS is caused most frequently by trisomy of chromosome 21, accounting for nearly 95% of cases [2]. Although DS is most frequently associated with cognitive disability, DS has higher incidences of multiple medical conditions spanning every organ system [3].

While DS itself is not considered a primary autoimmune disorder, individuals with DS have increased rates of various autoimmune diseases. Specifically, autoimmune thyroid disease, type I diabetes (T1D), and celiac disease occur at rates four to six times higher in persons with DS compared to the general population, raising questions about how and why DS and autoimmune disorders are so closely associated [4, 5]. It is presumed to be related to higher genetic activity from the triplicate copy of chromosome 21. The underlying molecular mechanisms of each autoimmune disorder and its relationship to chromosome 21 may provide a deeper insight as to why persons with DS are more prone to developing autoimmune diseases than the general population.

The purpose of this narrative literature review is to highlight the most prevalent and clinically significant systemic autoimmune diseases in individuals with DS. By exploring and highlighting how individuals with DS are at greater risk for developing a spectrum of autoimmune diseases, individuals can perhaps receive earlier diagnoses, treatments, and ultimately improved long-term health outcomes.

## Molecular Connection Between DS and Autoimmunity

Immune dysregulation is a feature of other well-known genetic conditions including Turner Syndrome, Kabuki Syndrome, 22q11.2 deletion syndrome, and Klinefelter Syndrome [5]. With a clear connection between chromosomal abnormality and immune dysregulation, it is hypothesized that the genetic differences underlying these syndromes alter the appropriate balance of gene expression, adversely impacting immune regulatory pathways, impairing apoptosis, and potentially leading to enhanced molecular mimicry and activation of autoreactive T or B cells by foreign-derived antigens [6].

A growing body of scientific research describes potential mechanisms for immune dysregulation in the setting of trisomy of chromosome 21. Important genes like the autoimmune regulator (AIRE) gene and four of the six interferon receptor subunits (which act as receptors for both type III interferon (IFN) ligands and cytokines interleukin-10, interleukin-22 and interleukin-26) are found on this chromosome, leading to an unbalanced gene dosage in individuals with DS [7]. Studies on chromosome 21 specific micro-ribonucleic acids (miRNAs), which are small endogenous non-protein-coding ribonucleic acids that can inhibit gene expression, show a possible contribution to a greater susceptibility to autoimmunity, as certain miRNAs including miR-99a, let-7c, miR-125b-2, miR-155, and miR-802 each decrease the expression of innate immune regulatory and anti-inflammatory genes [8]. One study demonstrated that individuals with DS can have an overexpression of IFN receptors, increased IFN hypersensitivity, altered expression of pro and anti-inflammatory cytokines, and impaired chemotaxis of mononuclear cells, all of which can impair innate immune system responses [5]. Other studies have identified that adaptive immunity in individuals with DS is also impacted by altered AIRE expression, thymic hypoplasia, and cortical atrophy, which can collectively contribute to higher rates of apoptosis in B and T cells, more resistant T helper cells to T regulatory (Treg) cell suppression, and an altered immunoglobulin (Ig) pattern of increased IgG but decreased IgM and IgE levels [5]. While there are many mechanisms by which autoimmunity could develop, the heterogeneity of pathways that can potentially be disturbed in persons with DS promotes a molecular milieu of immune dysregulation as opposed to frank autoimmunity.

Human leukocyte antigen (HLA) subtypes, which typically serve as a strong predictor for autoimmune diseases, have a complex role in individuals with DS. One study demonstrated that the major histocompatibility class II (MHC II) DQA1 0301 allele, which is controlled by regulatory immune genes on chromosome 21, is strongly overrepresented in those with DS and hypothyroid autoimmune disease [9]. Another study found that despite diabetes-associated HLA class II genotypes in children with DS and T1D being more prevalent compared to children without DS and T1D, the susceptibility of T1D in children with DS is only partially HLA mediated [10]. In fact, further studies raised that T1D associated with trisomy 21 actually has decreased HLA-mediated susceptibility compared to T1D in neurotypical children [11, 12]. Conversely, one study showed that seven of eight children with DS showed the CD-associated high-risk HLA DQA1 0501 DQB1 0201 genotype compared to 13 of 80 children with just CD, pointing to the idea that this CD-associated high-risk HLA genotype is more common in children with DS and CD compared to children with just CD [13]. However, another study showed that while persons with DS and CD have a significant increase in DQA1 0101 allele, persons with DS and CD compared to persons with only DS show no significant difference regarding serum HLA Class I antigens [14]. Furthermore, other studies show that children with DS and T1D were less likely to carry the highest risk genotype DR4-DQ8/DR2-DQ2 compared to children with just T1D, thus creating the need for further investigation into specific HLA haplotypes associated with both DS and autoimmunity [15].

Additional factors such as accelerated aging [16], chronic inflammation [17], decreased Vitamin D intake and levels [18, 19], and an unhealthy microbiota from poor dietary habits [20] could all be contributory to the increased likelihood of autoimmunity in individuals with DS, although they have been historically under-explored in this population [5].

## Thyroid Diseases

Multiple studies have demonstrated that individuals with DS are much more likely to develop thyroid dysfunction than the general population, with incidence rates over the lifespan as high as 63% [21]. While there are various causes of thyroid disease, autoimmunity as identified through the presence of thyroid autoantibodies is found in 13–34% of persons with DS [22]. The most frequently reported types of autoimmune thyroiditis include Hashimoto’s thyroiditis (HT) and Grave’s disease (GD) [4]. Prevalence rates of autoimmune endocrine conditions and other disorders discussed in this manuscript are presented in Table 1.

**Table 1:** Prevalence of autoimmune disorders in persons with Down syndrome

	Percentage of individuals with DS with condition	Percentage of neurotypical individual with condition	Citation
Endocrine			
*Hashimoto’s thyroiditis* *Grave’s disease* *Type I diabetes*	13–34% 0.66% 17%	3% 0.02% 0.4%	[4, 21] [4, 23] [15, 24]
Gastrointestinal			
*Celiac disease*	5.8%	0.5–2%	[25]
Rheumatoid			
*Arthropathy*	0.8–2%	n/a	[26–28]
Dermatologic			
*Alopecia areata* *Hidradenitis suppurativa* *Vitiligo*	27.7% 2.1% 4.4%	2% 0.3% 0.05–1.55%	[29] [30] [29]
Neurologic			
*Moyamoya* *DSRD*	0.001% unknown	0.0001% n/a	[31] [32, 33]

Atopic dermatitis prevalence in non-DS: 32,412,646

Vitiligo in non-DS: 34,787,670

The prevalence of hypothyroidism in newborns, from birth until 6 months of age, is 20% and it was found that 50% of young children with DS will develop some form of thyroid dysfunction, including HT, by the time they enter adulthood [34–36]. HT is a chronic autoimmune hypothyroid disorder that destroys thyroid cells, leading to an improper balance of thyroid hormones [34, 35]. One study found the prevalence of HT in persons with DS to be estimated around 36.6% in an age group of two- to seventeen-year-old persons [37]. Although HT typically affects females more often than males at a ratio of at least 10:1, those with DS and HT present with an even sex distribution of 1:1 [38]. This even sex distribution is atypical in autoimmune literature and may support the genetic basis for disease in this population. Individuals with DS experience HT at an earlier age of onset and have lower levels of thyroid autoantibodies such as thyroid peroxidase (TPO) antibodies, making diagnosis challenging and clinically important [39]. Unlike neurotypical individuals, there is rarely a family history of thyroid dysfunction that often serves to increase screening of individuals on a clinical basis, although guidelines have improved the screening for these disorders possibly based on the higher awareness of the increased association between thyroid disorders and DS by physicians [39].

The pathogenesis of HT is caused by the production of pathogenic antithyroid antibodies from B cells, which bind to various antigens within thyroid tissue including TPO, Thyroid Stimulating Hormone Receptor (TSH-R), and thyroglobulin [34]. Once the thyroid cells are bound and marked, these antibodies can not only block thyroid cell activity but also fix complement, induce oxidative stress, and stimulate cytotoxic T cells to cause apoptosis, resulting in antibody-mediated cell death [40]. This destruction from the infiltration of lymphocytes can lead to fibrosis and atrophy of the thyrocytes resulting in long-term insufficient production of thyroid hormone.

Clinical manifestations of HT include an array of physical changes in the patient, such as obesity, fatigue, constipation, and intellectual impairment which make clinical differentiation in individuals with DS difficult [34, 35]. For this reason, the American Academy of Pediatrics recommends screening for HT at 6 months, 1 year, and every year after that for life to identify endocrinologic dysfunction early to optimize treatment [41]. Diagnosis of HT includes testing for antithyroid antibodies in conjunction with thyroid-stimulating hormone (TSH) and level of thyroxine (T4), which are typically elevated and reduced respectively [34]. While treatment is merely to counteract symptoms rather than cure hypothyroidism, early thyroid hormone replacement medication like synthetic levothyroxine (LT4) and proper dietary management have been associated with maximized growth and intellectual development, thus preventing severe hypothyroidism and its resulting susceptibility to physical and intellectual defects [23].

GD is a chronic autoimmune hyperthyroid disorder that over activates the thyroid cells to overproduce thyroid hormone and accelerate bodily functions and metabolic demand [42]. This disorder represents the main cause of hyperthyroidism and has a prevalence of 0.66% in those with DS compared to 0.02% in the general population [43]. GD can occur as early as birth, but commonly presents in late childhood or early adult life in persons with DS [44]. GD in persons with DS differs diagnostically compared to the general population in that there is a younger age at diagnosis, a lack of female predisposition, a propensity to convert to GD from HT, less severe clinical course and decreased rates of remission [38].

Unlike HT, the pathogenesis of GD involves the presence of TSH receptor IgG immunoglobulins (TRAb) from B cell clones that infiltrate the gland and mimic the effects of TSH on thymocytes, causing autonomous production of triiodothyronine and thyroxine [42]. Specifically, this autoimmune reaction involves a large Th1 immune response in which TRAbs pathogenetically activate adenylate cyclase which subsequently produces cyclic adenosine monophosphate, propagating thymocyte proliferation, thyroid gland growth, and increased production of T3 and T4. With the increased levels of T3 and T4, TSH secretion is inhibited via negative feedback, leading to the characteristic decreased TSH levels in individuals with GD [42–45].

Clinical manifestations of GD in those with DS include, but not limited to, fatigue, restlessness, weight loss, and diarrhea—all of which are similarly seen in persons with GD and without DS. Consequences of untreated GD are goiter, Grave’s orbitopathy, myxedema, congestive heart failure, atrial fibrillation, neurological defects, and osteoporosis, thus reducing the quality of life and potentially increasing mortality of those afflicted [42]. Even though GD is rarer than HT in patients with DS, the American Academy of Pediatrics still recommends screening for GD at 6 months, 1 year, and every year after that for life, similar to HT screening protocols [41].

Regarding treatment, there is no difference in management amongst persons with DS and those without. Initial therapy involves antithyroid drugs (ATD) which normalize thyroid function [46]. Other therapies include radioactive iodine and thyroid surgery, but radioactive iodine is typically preferred over surgery in patients with DS due its less invasive nature and patients having specific facial anomalies that can cause airway obstruction and difficult anesthesia during surgery although evidence is anecdotal [25]. In those with a history of DS and neoplasia (who have received radiotherapy or chemotherapy), use of radioactive iodine is not directly contraindicated although should be discussed with the medical care team.

## Celiac Disease

Celiac disease (CD) is an autoimmune gastrointestinal disorder triggered by immunologic reactions to gluten, a protein found in wheat, barley, and rye. While the prevalence of CD in Europe and worldwide is approximately 1–2% and 0.5–1%, respectively, studies show 5.8% of individuals with DS also have CD, representing a marked enrichment in this population [47].

The pathogenesis of CD involves an inappropriate adaptive immune response in which antibodies to tissue transglutaminase are formed to attack the small intestine and cause inflammation, villous atrophy, and difficulty absorbing nutrients [48]. Specifically, gluten derived proteins induce a substantial CD4 and CD8 T lymphocyte response, yielding elevated levels of pro-inflammatory cytokines and clonal expansion of B lymphocytes, which subsequently secrete anti-gliadin and anti-tissue transglutaminase antibodies [49]. These antibodies and gliadin specific CD8 T cells locate themselves in the sub-epithelial or epithelial brush border region of the small intestine [50].

Clinical manifestations of CD include, but are not limited to, abdominal pain, anemia, inadequate nutrition, chronic diarrhea, vomiting, abdominal distension, and fatigue [51]. More rarely, ataxia, cognitive impairment or neurodevelopmental stagnation, and seizure can also be associated with CD [52, 53]. These symptoms complicate the diagnosis of CD in persons with DS, as growth impairment, anemia, intermittent diarrhea, epilepsy, neurodevelopmental delay, and constipation are frequently encountered in persons with DS Without CD. Thus, increased clinical vigilance and practical awareness of these characteristic symptoms of CD are required to ensure proper and timely diagnosis in persons with DS.

Diagnosing CD typically involves HLA testing for HLA-DQ2 and HLA-DQ8 or detection of autoantibodies like anti-gliadin or anti-endomysial antibodies, with possible confirmation using histological analysis of duodenal biopsies [51]. Current guidelines published by the American Academy of Pediatrics recommends serologic screening for CD only in symptomatic patients with DS and a gluten-containing diet, beginning at age one and following up at every preventative care visit [54]. Because CD can develop at any age, repeat testing in adulthood is often warranted, even when prior testing was negative.

The only established treatment for CD regardless of DS status involves a complete and lifelong restriction to a gluten-free diet [55]. Avoidance of all gluten-containing foods as well as gluten-containing lifestyle products such as hygienics or cosmetics is necessary to decrease the severity of clinical symptoms and reduce the risk of severe complications [24].

## Type 1 Diabetes

Type 1 diabetes (T1D) is an autoimmune disorder of the pancreas wherein there is destruction of insulin producing Islet of Langerhans beta cells. In children with DS, 17% are diagnosed by age 2 years compared to 0.4% in the general population [15, 56, 57]. The age of onset of T1D in persons with DS is also skewed towards younger, often pre-pubertal, ages [58, 59].

The pathogenesis of T1D involves autoreactive CD4 T cells that bind beta cell components including insulin B chain peptide, glutamic acid decarboxylase, and other beta cell secretory granules, identifying them as foreign antigens. These CD4 cells not only erroneously stimulate antigen presenting cells to create high affinity autoantibodies but also induce a cytokine cascade that helps CD8 T cells to perform cell-mediated attacks on beta cells [60]. Once damage of beta cells begins, a positive feedback loop ensues in which more cytokines are released leading to further recruitment of immune cells for further beta cell destruction [61]. After 80% or more of insulin producing cells have been destroyed, clinical signs and symptoms of T1D present.

Clinical features of T1D do not largely differ between those with and without DS. These include polydipsia, polyuria, fatigue, weight loss, and vision changes which can occur acutely or over several months in some circumstances. Long term complications of untreated T1D include blindness, peripheral neuropathy, diabetic ketoacidosis, nephritis, and cardiovascular disease, highlighting the importance of early identification and intervention. The American Diabetes Association recommends that screening for T1D in individuals with or without DS should begin when one or more signs or symptoms of diabetes is present although asymptomatic testing is not currently advised [62]. Positive values indicating possible T1D for these tests include a random blood sugar test level of 200 mg/dL or higher, a glycated hemoglobin A1C test of 6.5% or higher, and a fasting sugar test of 126 mg/dL or higher [26].

Treatments for patients with DS and T1D differ from those with just T1D in that simpler insulin regimens that require fewer applications a day are given to improve adherence and make administration more convenient for caregivers [38]. Interestingly, one study showed that despite developing diabetes earlier, individuals with DS and T1D achieved better metabolic control with lower insulin doses compared to persons without DS, possibly owing to a simpler lifestyle and use of structured routine [27]. The lower dose of insulin required in a regimen for persons with DS may suggest that T1D in patients with DS may be associated with incomplete beta-cell function loss although this hypothesis has not been validated.

## Arthropathy and Other Rheumatoid Conditions

Although not traditionally considered autoimmune, inflammatory conditions such as arthropathy seem to be more prevalent in individuals with DS. Estimates of the prevalence of arthropathy in individuals with DS (ADS) is approximately 8.7–12.2 in 1000; three times higher than the general population [28, 63, 64]. Regional differences may yield even higher rates with one Irish study reporting the presence of ADS as 33 of 503 children with DS, equating to a prevalence of 20 per 1000 individuals [64]. Interestingly, despite the elevated risk for ADS, the mean time to diagnosis for juvenile idiopathic arthropathy (JIA) in the general population was 3 months, while the mean time to diagnosis for ADS was markedly longer at 3.3 years [65, 66]. This delay can be attributed to a number of factors including poor awareness by providers and caregivers about ADS, a gradual functional loss over time attributed to behavioral problems, delay in motor milestones attributed to intellectual disability, limited verbal skills, and differences in expression and tolerance of pain [64]. Delays in diagnosis could be adversely affect motor function making a high index of suspicion critical in this population [66].

While the exact pathogenesis of ADS is unknown, studies show that compared to individuals with JIA, those with ADS show an expansion of IgM memory B cells, decreased proportion of transitional B cells, increased TNF and INF-γ responses by CD8 T cells, increased TNF and IL-17A responses by CD8 T cells, significant expansion of Th1/Th17 cells, and reduced Treg cells, collectively suggesting immune dysregulation in individuals with DS [64, 67]. In spite of their differences, ADS appears to have a shared pathogenesis with JIA, which involves immunologic changes with abnormal activation of immune cells, production of proinflammatory cytokines, and autoantibody formation that all target joint cells and cause joint destruction [68]. These may indicate that DSA is a unique subtype of JIA in individuals with DS, intrinsic to the unique immune regulatory signature observed in individuals with DS or that DSA and JIA exist on a clinical and pathophysiologic spectrum. In the setting of a growing expansion of our knowledge regarding rheumatoid and inflammatory processes in individuals with DS, it is possible that more traditional autoimmune mechanisms may be elucidated on ADS in the near future.

Clinical differences between arthritis in the general pediatric population and ADS are that ADS is typically rheumatoid factor (RF) and antinuclear antibody (ANA) negative, with an aggressive polyarticular disposition favoring the small joints of the wrists and hands [64]. Additionally, children with DS show a high proportion of erosive changes at diagnosis along with a greater degree of permanent joint damage, suggesting that arthropathy in persons with DS is more aggressive than in the general population [64, 69]. Moreover, despite most oligoarticular JIA having a sex predilection toward females, ADS shows no sex bias, indicating the primary cause of immune dysregulation may be uniquely driven through trisomy of chromosome 21 [69]. Finally, compared to JIA, ADS showed a greater elevation of erythrocyte sedimentation rate (ESR) and a decreased incidence of uveitis [69].

Without formal guidelines on screening or monitoring for ADS, delayed diagnosis and permanent joint damage is prevalent [69]. Thus, clinical vigilance and enhanced awareness must be exercised to ensure a correct and timely diagnosis. One study suggests that an annual musculoskeletal assessment involving X-ray be implemented in physician guidelines in individuals with DS who are symptomatic for joint pain with an MRI using gadolinium contrast used to confirm the diagnosis of arthritis, although no formal guidelines or standards or care exist at present [64].

While there is no current guidance on optimal treatment for ADS, many physicians suggest using nonsteroidal anti-inflammatory drugs, oral or intraarticular steroids, and disease modifying anti-rheumatic drugs with methotrexate being a common second line therapy despite many individuals with DS showing intolerance to the drug [29]. Additionally, biologic therapies such as TNF inhibitors have been used such as abatacept or tocilizumab, yet these therapies still show some level of ineffectiveness as a large portion of patients have at least one change in biologic therapy [63]. Ultimately, a nuanced and personalized approach must be taken in cases of ADS, and any suspected inflammatory or autoimmune rheumatologic condition, although advances in our understanding of immune function in persons with DS will hopefully spur new advances in the treatment of this condition.

Interestingly, other autoimmune rheumatologic conditions appear to be of similar prevalence in individuals with DS. There have been no large-scale reports of an increased prevalence of disorders such as systemic lupus erythematosus [30], Sjogren’s syndrome, ankylosing spondylitis, dermatomyositis, or systemic sclerosis. It remains unclear if these disorders are under-reported in individuals with DS or if they are not upregulated to the point of frequent reporting in medical literature. Importantly, some rheumatologic conditions such as mixed connective tissue disorder may be even more challenging to diagnose in individuals with DS given their multi-system involvement and overlap between core symptoms (e.g., muscle weakness, esophageal dysmotility, and leukopenia) [70] and commonly observed findings in otherwise healthy individuals with DS.

## Dermatologic Disease

There are several autoimmune and inflammatory dermatologic disorders that arise in persons with DS including alopecia areata (AA), hidradenitis suppurativa (HS), and vitiligo. One questionnaire-based study reported that 56% of young adults with DS had dermatologic disease [71]. A systematic review found the prevalence of AA in persons with DS to be 7.4% with a mean age of onset of 7 years old, a mean duration of 2.7 years, and a recurrence of 27.7% [72]. This is in contrast to a 2.1% lifetime risk in individuals without DS [73]. This study also found the mean prevalence of vitiligo in patients with DS to be 4.4%. Moreover, HS had a prevalence of 2.1% compared to 0.3% in the general population and presented at a younger age [74]. Interestingly, these autoimmune and inflammatory skin conditions can be complicated by the fact that chronic immune dysregulation may promote microbial growth and create cutaneous dysbiosis and frequent skin infections, which are also common in this population [75].

The pathogenesis of autoimmune and inflammatory dermatologic conditions in persons with DS is heterogenous and complex. The most well described amongst these conditions is AA which is caused by a loss of immune privilege of the hair follicle, an upregulation of the immune inflammatory pathway, and the subsequent autoimmune destruction of the follicle [76]. Specifically, the disruption of the hair follicle’s immune privilege involves plasmacytoid dendritic cells, natural killer and T cells along with IFN-γ and IL-15 collectively inducing hair follicle destruction and the resulting hair loss [77]. Because chromosome 21 encodes four of six IFN receptor subunits and those with DS experience IFN receptor overexpression in various cell types, some studies suggest that the hyperactivity of IFN can enhance induction of downstream JAK/STAT signaling, mechanistically leading to the loss of the hair follicle in AA [77–80]. Thus, certain new experimental treatment strategies for AA involve JAK-STAT inhibitors which downregulate the JAK/STAT pathway and potentially block the pathogenesis of AA [81, 82].

Clinical features of AA can vary from small, well circumscribed areas of hair loss to total absence of hair on the body or scalp [31]. However, in persons with DS, studies show that the localized type is much more common with discrete, non-scarring patches on the scalp being the most frequent location [83]. In regard to the diagnosis of AA, after providers note at least 2–5 cm in diameter of hair loss, they should ensure that thyroid screening is up to date, as autoimmune conditions can run concomitantly [78]. While there is no definitive cure for AA, treatment for limited AA includes corticosteroid injections or topical steroids to the area of hair loss [84]. Additionally, minoxidil can be used to increase hair growth via acceleration of the natural hair cycle [85]. While AA is not a serious condition regarding health risk, this disease can lower self-confidence and reveal personal insecurities in body image, thus requiring provider and caregiver emotional support.

While the research in autoimmune dermatologic conditions has accelerated in the past decade, existing published knowledge has been relatively restricted to AA. At present, there are phase II immunotherapy clinical trials running in the United States and it is hopeful that our knowledge will continue to expand in the near future.

## Neurological Disorders

Disorders of the nervous system were not traditionally thought to be of autoimmune etiologies although emerging evidence over the last decade has indicated that the brain and cerebrovascular system may also be a target of immune dysregulation observed in other organ systems of persons with DS. These disorders are rare and are still under investigation with regard to the exact mechanism and immunotherapeutic interventions.

Moyamoya disease (MMD) is a chronic occlusive cerebrovascular disorder that involves the progressive stenosis of the proximal intracranial arteries and the development of occlusion in collateral vessels [86, 87]. Despite having a worldwide prevalence of 10.5 per 100,000 individuals, MD disproportionately affects persons with DS with a prevalence of 145 per 100,000, representing a 26-fold increase compared to the general population [88]. Interestingly, one study demonstrated that the prevalence of autoimmune disease in persons with DS and MMD was 57.7%, while the prevalence of autoimmune disease in idiopathic MMD and DS alone was 20.3% and 35.3%, respectively [32]. Furthermore, studies show that increased thyroid autoantibodies and elevated thyroid function are strongly associated with a more aggressive disease course in MMD in children [33]. Despite the unknown etiology of MMD, the association between MMD, DS, and autoimmunity points to the potential that the development of MMD has an autoimmune or inflammatory component.

Clinical features of MMD in persons with DS do not largely differ from the general population, with a range of symptoms from transient ischemic attack to permanent unilateral neurological defects in the form of stroke, seizures, and headaches/migraines [89, 90]. Interestingly, the average age of diagnosis of MMD in the general population is 7.1 years compared to 9.3 years in persons with DS, suggesting that there is greater difficulty in detecting MMD in persons with DS and that transient neurologic symptoms of MMD in persons with DS may be attributed to other coexisting morbidities like cardiac anomalies or independent seizure disorders [89]. Guidelines created by the Research Committee on Moyamoya Disease suggest that the diagnosis of MMD in persons with DS primarily requires cerebral angiography or, if unavailable, MRI neurovascular imaging, with treatment entailing surgical revascularization [91]. At present, there is no guideline or recommendation regarding screening asymptomatic individuals which is problematic as nearly 70% of individuals with DS and MMD will present with stroke [92].

Down syndrome regression disorder (DSRD) is an emerging neurocognitive disorder of unclear etiology reported in young persons with DS between ages 10 and 30 years [93, 94]. Symptoms include a subacute loss of previously acquired developmental skills in the areas of language, communication, cognition, executive function, behavioral, and adaptive skills [93–98]. Other symptoms can include psychiatric manifestations, bradykinesia, catatonia, and rapid onset insomnia. DSRD can be severe and significantly impact both the quality of life and autonomy of persons with DS. There are no studies evaluating the exact incidence of this condition although it is presumed to be quite rare.

This disorder was previously presumed to be psychiatric or even neurodegenerative in origin although emerging evidence has identified evidence of cerebrospinal fluid abnormalities in persons with DSRD indicative of neuroinflammation [95]. The presence of restricted oligoclonal banding and elevated IgG indices in the cerebrospinal fluid indicate a potential B-cell mediated process although T-cell and interferon driven dysregulation is also suspected given the heterogeneity of findings.

Therapeutic interventions for this condition are broad and have ranged from antipsychotics to immunotherapy [99]. A minority of individuals with DSRD may have a neuroinflammatory etiology to the disease, confirmed by the presence of abnormal neurodiagnostic studies and dramatic immunotherapy responsiveness in some patients [93, 95, 100, 101]. While immunotherapy provides a tool to rapidly reverse this clinical syndrome, guidance on dosing and duration of therapy remains unclear.

## Potential Mechanisms to Explain Enhancement of Specific Autoimmune Conditions in Down Syndrome

The exact mechanisms by which trisomy of chromosome 21 causes immunologic dysregulation remain unclear. Individuals with DS are established to have immune regulation issues as previously detailed although select enhancement of certain autoimmune and inflammatory conditions is of great significance and may potentially point towards more refined biochemical and immunologic investigation in the future. At present, three interconnected hypotheses regarding abnormalities intrinsic to persons with DS have been proposed to account for enhanced autoimmune disease prevalence in this population.

### Interferon Dysregulation

**Fig. 1:**
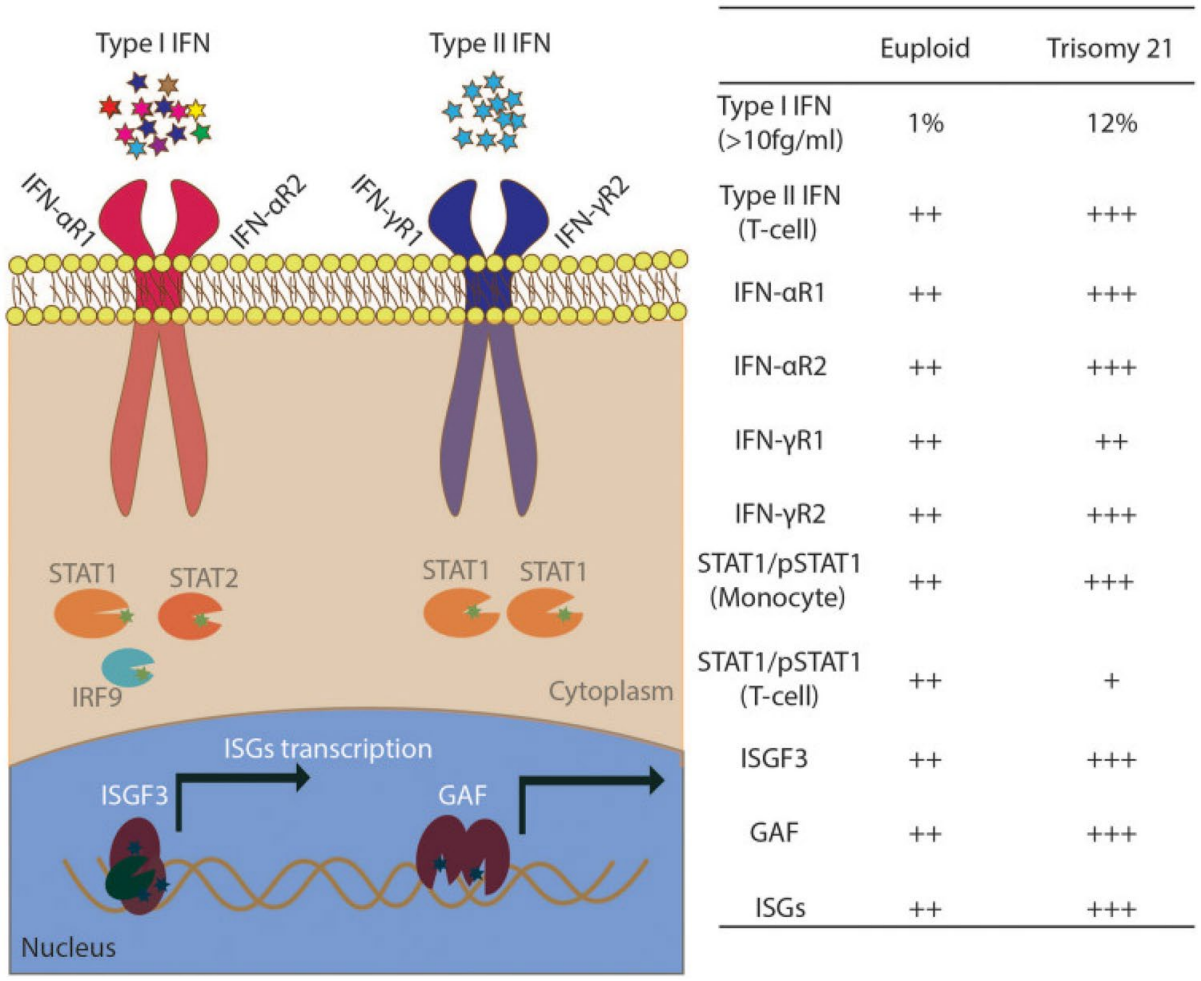
Upregulation of interferon receptor genes in trisomy 21. Reprinted with permission from Chung et al. [112]

Interferon dysregulation in individuals with DS is well-established and produced by trisomy of chromosome 21 which contains a cluster of four out of six interferon receptor (IFN-R) genes: IFNAR1, IFNAR2, IFNGR2, and IL10RB (Fig. 1) [102], These genes have been previously shown to have variable expression in multiple cell types in individuals with DS and can account for baseline interferon activity and intermittent “variable” interferon activity, as would be seen in an individual having increased interferon signature in the setting of having a viral illness [103]. These unique interferon signatures have been associated with a variety of different autoimmune diseases and may specifically enhance the likelihood of the development of HT [104–106], CD [49], type 1 diabetes [107, 108], and a variety of dermatologic conditions [109–111]. Although neurological conditions in DS remain less studied, individuals with DSRD appear to have radiographic signatures consistent with increased interferon activation in the bilateral basal ganglia [101]. Dysregulated interferon and subsequent activation of JAK/STAT pathways may yield a variety of downstream regulators including cytokines, chemokines, and other immunologically reactive cells. As such, the interferon overactivation hypothesis may be a starting point for downstream immune regulation in individuals with DS.

### Cytokine Overactivation

The regulatory mechanisms in the JAK/STAT molecular cascade are complex and diverse and are uniquely associated with cytokine activation [113]. Although multiple negative control mechanisms such as receptor internalization, negative feedback loops (via cytokine signaling suppressor proteins), and direct protein inhibition are in place in neurotypical individuals, the excessive activation of these pathways in persons with DS inevitably produce excessive cytokine release in response to a variety of antigen exposures [113–116].

Cytokines, while not in and of themselves causative of autoimmunity, have been linked to pathology observed in a variety of autoimmune conditions [117, 118]. Immunologic cell line activation is another prominent potential driver of autoimmune disease and another potential reason for the limited set of autoimmune conditions in individuals with DS. Multiple studies have identified the potential impact of cytokine activation on both B-cell populations [119–122] and T-cell populations [123–125]. In addition, activation of natural killer cell pathways may further potentiate the cellular destruction capacity of overactive cytokine signalling [126, 127]. Ultimately, the sheer number of cytokines and multifaceted activity of each one makes determination of pathogenicity challenging. As such, differentiating the overlap of cytokines and downstream cellular activation in each individual with DS is complex but a ripe area for future investigation, particularly with regard to B-cell function and immunoglobulin generation.

### Increased Propensity for Pathologic Autoantibody Generation

**Fig. 2:**
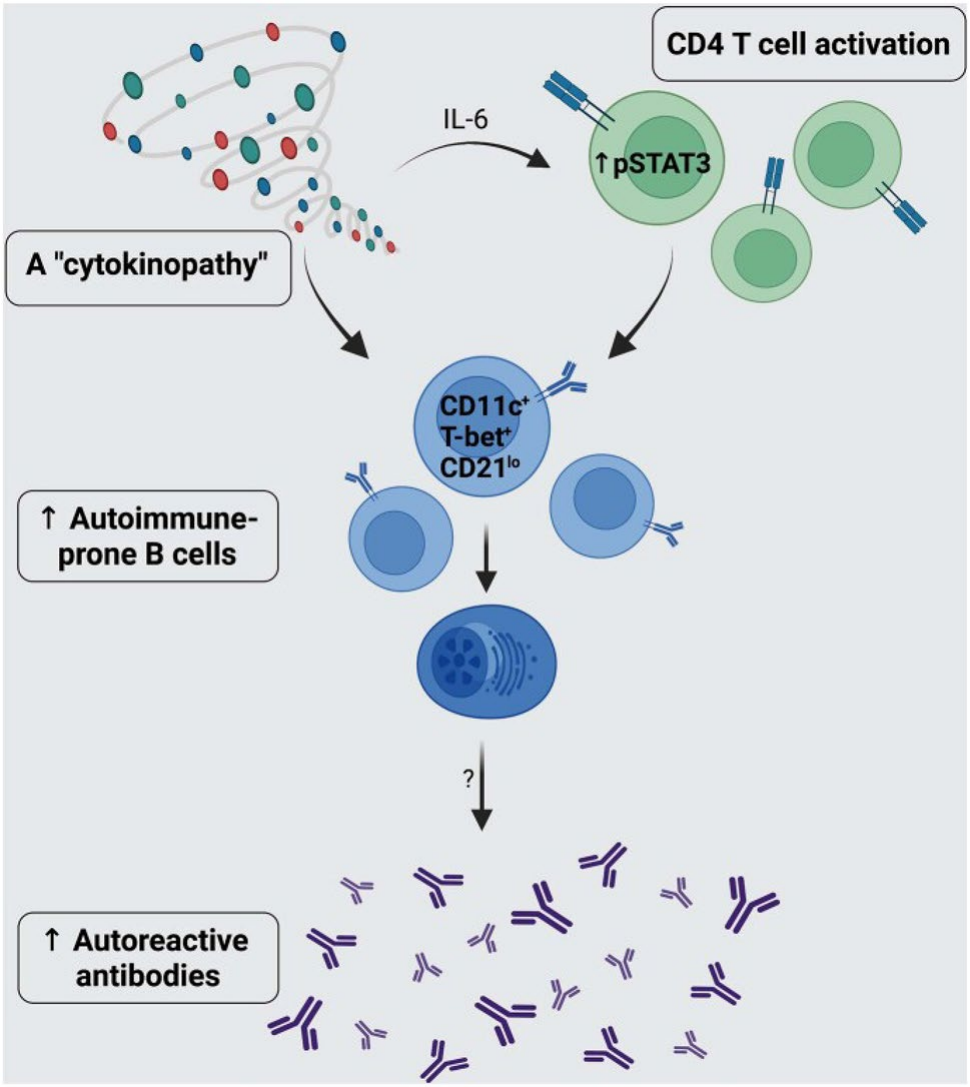
Breaking immune tolerance in Down syndrome: a triad of cytokines, activated T cells and CD11c + B cells. Reprinted with permission from Malle et al. [67]

Recent studies have detailed that baseline cytokine milieu and enhanced CD11c + B cell frequency may drive both an increased likelihood of pathologic autoantibody generation but also be directly tied to symptomatic autoimmune disease [67, 102]. As graphically demonstrated in Fig. 2, individuals with DS may experience enhanced direct activation of CD11c + and indirect activation via CD4 + T cells with upregulated pSTAT3 via cytokine overactivation. Interestingly, these pathways may be bidirectional and actually feed forward into one another as T-cell differentiation and activation have previously been documentation as being positively associated with one another [128] in the context of a limited ability of T regulatory cells to suppress CD8 + and CD4 + effector cells.

From a pathway perspective, this increased likelihood of antibody generation is logical given the role that overactivation of interferon receptors has on enhanced JAK/STAT signaling [102]. However, an important feature of this increased likelihood, particularly in the context of developing chronic autoimmune disorder, may be the enhanced potential for molecular mimicry. In this setting, it would be hypothesized that the increased interferon and cytokine activation present in individuals with DS responding to a foreign antigen (e.g., viral proteins) would be more likely to generate pathologic antibodies through the above-referenced mechanisms [67, 129]. In addition, epitope spreading may be propagated in an accelerated fashion given both the level of chronic inflammation present in individuals with DS but also the frequency of pathologic autoantibody binding, as observed in other chronic inflammatory conditions [130, 131]. This may explain the high rate of having multiple autoimmune diseases in persons with DS [32, 132].

From a phenotypic standpoint, the activation of interferon, cytokines, CD4 + cells, and CD11c + cells may partially explain the variety of different autoimmune conditions observed in persons with DS. Each pathway represents a unique opportunity to develop an autoimmune condition, or conversely, activate downstream pathways that may similarly induce an autoimmune disease of a different organ system. This may also explain why therapeutic interventions against JAK/STAT pathways, IL-6 blockade, or B and T cell modulation (IVIg) have been demonstrated as very effective in persons with DS [128]. The complex interrelationship between dysregulated pathways intrinsic to persons with DS may produce polyfactorial etiologies to the autoimmune conditions observed. It is possible that these pathways make be more or less active at different ages in persons with DS and be linked to some of the non-autoimmune neurodegenerative conditions observed in this population as well [133].

## Conclusion

There is increasing evidence indicating that trisomy of chromosome 21 may be the driving factor in increasingly definitive clinical associations between autoimmunity and DS. Further investigation into the molecular and genetic mechanistic pathways of these autoimmune diseases in persons with DS will hopefully lead to more targeted and personalized interventions in the future.
